# Western Ophthalmologic and Otorlaryngologic Association

**Published:** 1899-01

**Authors:** Thomas A. Woodruff

**Affiliations:** Secretary; Chicago


### Society Notes.

### Western Ophthalmologic and Otorlaryngologic Association.

CHICAGO, December 16, 1898.

MY DEAR DOCTOR: The fourth annual meeting of the Western Ophthalmologic and Otolaryngologic Association will be held in New Orleans February 10th and 11th, 1899.

Already a large number of Ophthalmologists and Otologists have promised to attend, and we shall be glad to add your name to the list.

As the Madri Gras takes place in New Orleans on the 13th and 14th of February, it will be necessary to reserve hotel accomodations at an early date. For information concerning hotel rates, etc., kindly address Dr. Wm. Scheppegrell, 124 Baronne Street, New Orleans, La.

Arrangements have been made with the Illinois Central Railroad to transport members of the above association from Chicago to New Orleans and return, going via Memphis and Jackson, Miss., returning via Baton Rouge and Vicksburg to Memphis, making a diverse route from that point. This will be appreciated by visitors to the South as it enables them to pass many historic points en route south, and the unsurpassed sugar and rice producing district between New Orleans and Baton Rouge, in addition to Vicksburg and other war-famed spots, north bound.

The intention is to charter a Pullman sleeper to be used en route and while in New Orleans (same being tracked at the magnificent and centrally located passenger station of the Central.) The sleeper will cost $45.00 per day from time of departure until returned, and fifteen tickets will be required by the railroad company to haul the car, giving us the exclusive use of it. For this reason we desire to bring as many of the members together in Chicago as possible. A standard sleeper will accommodate twenty-six people, giving an entire berth to each. This will reduce the expense to a minimum, and make hotels unnecessary, meals being taken en route in cafe car and at railroad eating houses, and at the famous restaurants of New Orleans while there, the hotels being crowded at this Mardi Gras season.

The low rate of $25.00 for round trip from Chicago is always in effect for the carnival season. Tickets will be on sale several days in advance, with about twenty days' return limit; thus members can make the trip at a nominal cost.

Attend the meeting, see the quaint old French city at its gayest, and enjoy the beauties of a southern trip at a time of year when most appreciated.

Leaving Chicago at 5:45 p. m., Wednesday, February 8th, we reach Carbondale at about midnight, where members from St. Louis can join, arriving in Memphis in the early morning, where those from Louisville, Cincinnati and the east will arrive at practically the same time, bringing members from many States together while yet nearly 400 miles from the Crescent City, which is reached at 7:45 p. m., only twenty-six hours from Chicago.

An early reply is desired from those who desire to join us, so that we can make berth reservations and complete the details to the mutual advantage of all.

Yours very truly,

THOMAS A. WOODRUFF, Secretary.

